# Multifunctional Flame-Retardant, Hydrophobic, and Antifungal Boron–Silane Synergistic Coating for Archival Packaging Materials

**DOI:** 10.3390/polym18172168

**Published:** 2026-09-05

**Authors:** Juanli Wang, Qingyang Xie, Ye Tian, Jiayao Tian

**Affiliations:** Key Laboratory of Archaeological Exploration and Cultural Heritage Conservation Technology, Ministry of Education, Institute of Culture and Heritage, Northwestern Polytechnical University, Xi’an 710072, China; 18735967040@163.com (Q.X.); tianye08192024@163.com (Y.T.); 18791731988@163.com (J.T.)

**Keywords:** kraft paper, archival packaging materials, flame retardation, antifungal activity

## Abstract

Kraft paper, as a typical archival packaging material, is prone to microbial erosion, flammability, and moisture-induced degradation during long-term preservation. In this work, multifunctional archival packaging paper with integrated flame retardancy, antifungal activity, and hydrophobicity was successfully fabricated via the modification of boron-based flame retardants (borax, BX, and disodium octaborate tetrahydrate, DOT) and silane hydrophobic agent (octadecyltrimethoxysilane, ODTMS). The structure and performance of the modified kraft paper were systematically characterized by the limiting oxygen index (LOI), the vertical burning test, cone calorimetry (CCT), thermogravimetric analysis (TGA), X-ray photoelectron spectroscopy (XPS), contact angle measurement, and the antifungal test. The optimized modified sample exhibited superior comprehensive properties. The LOI value increased from 20.6% for the original kraft paper to 37.5%. TGA revealed that the char residue of the optimized modified sample at 750 °C reached up to 55.35%, much higher than that of the original kraft paper, accompanied by marked decreases in the heat release rate, total heat release, and smoke production. Meanwhile, ODTMS endowed the modified sample with hydrophobic properties, with a water contact angle of approximately 130°. Antifungal tests demonstrated that the modified kraft paper displayed obvious inhibitory effects against eight fungal species, including *Penicillium citrinum* and *Aspergillus flavus*. Overall, the modification strategy proposed in this study is simple, eco-friendly, and low-cost, achieving multifunctional protection of archival packaging materials. This work is expected to provide novel materials and technical support for the long-term safe preservation of paper cultural relics and archives.

## 1. Introduction

Paper archives are important carriers of human civilization [1]. During long-term storage, paper is susceptible to various factors such as light, temperature, humidity, air pollutants, and microorganisms [2,3], leading to problems including degradation, discoloration, and performance deterioration [4,5,6]. Furthermore, flammability is a critical weakness, and numerous precious documents have been destroyed in fires [7,8], resulting in immeasurable losses. To extend the service life of documentary materials, the protection of paper archives is urgently needed.

Preventive conservation, as a core strategy for extending the lifespan of paper archives, emphasizes reducing the risk of damage through early intervention [9]. Archival packaging materials, serving as the “first line of defense” in direct contact with documents, directly determine the effectiveness of preservation. Currently, specialized companies abroad (e.g., Klug Company in Germany) have developed and produced dedicated acid-free paper as packaging materials for museum cultural relic protection [10]. However, the high import costs limit their large-scale application in museums, archives, and other fields in China. Meanwhile, unmodified acid-free paper has relatively singular functionality, focusing solely on anti-aging preservation, and possesses inherent limitations; namely, it lacks properties such as hydrophobicity, flame retardancy, and antibacterial performance. Si et al. [11] introduced STA to transform the hydrophilic smoke-suppressing flame-retardant Mg(OH)_2_ into a hydrophobic type (with the contact angle increased from 0° to 150°) while incorporating the highly hydrophobic filler DTMGP, which has a flame-retardant effect on the char-forming performance of PDA, successfully preparing an environmentally friendly, superhydrophobic, flame-retardant recycled paper. Mai et al. [12] prepared nanomodified archival boxes to inhibit mold growth by regulating microenvironmental carbon–nitrogen levels. They screened inorganic substances including Na_2_B_4_O_7_·10H_2_O, H_3_BO_3_, Na_2_B_8_O_13_·4H_2_O, C_6_H_7_KO_2_, nano-Mg(OH)_2_, nano-Zn_3_B_2_O_6_, and polyacrylamide for antimould activity using five fungal strains: *Aspergillus niger*, *Aspergillus flavus*, *Penicillium citrinum*, *Cladosporium* sp., and *Alternaria alternata*. Motelica et al. [13] introduced ZnO nanoparticles into cellulose paper to achieve dual antibacterial and antifungal effects.

Various coating strategies have also been reported to improve the fire safety of kraft and cellulose-based paper materials. Chelating self-assembly of phytic acid–chitosan–metal complexes can construct bio-based flame-retardant layers on kraft paper, enhancing the limiting oxygen index and char-forming capacity while maintaining mechanical properties [14]. Electrostatic-adsorption-constructed core–shell ammonium polyphosphate-based flame retardants have also been applied for paper modification, achieving significant reduction in peak heat release rate and total heat release [15]. Phytic acid-based interfacial assembly systems, such as phytic acid/ammonium dihydrogen phosphate composites, impart self-extinguishing performance to cellulose paper via condensed-phase carbonization [16]. Graphene-oxide-branched-polyethylenimine diffusion-driven self-assembly coatings endow packaging paper with flame-retardant and fire-sensing capability [17]. Moreover, spray-deposited composite coatings consisting of lignin, phytic acid and sodium silicate realize silicon-phosphorus synergistic flame retardancy for cellulose paper [18]. However, most existing modification techniques, whether developed for archival-relevant substrates or conventional packaging paper, only achieve single or dual functional optimization. It remains challenging to integrate high-efficiency flame retardancy, durable hydrophobicity, and broad-spectrum antifungal performance into a single paper substrate. Pure silane treatment endows paper with high hydrophobicity but fails to provide effective flame-retardant and antifungal capabilities, serving only as a moisture-resistant barrier. Metal-based modifiers such as ZnO nanoparticles improve microbial resistance but exhibit limited flame-retardant effects; their practical application is constrained by particle agglomeration, high cost, and poor compatibility with cultural relics. Silicate-based coatings enhance thermal stability but possess weak antifungal performance and require additional biocides, increasing processing complexity. Although binary functions (hydrophobic–flame-retardant and hydrophobic–antibacterial) have been realized in previous studies, few archival packaging systems can simultaneously achieve condensed-phase flame retardancy, broad-spectrum antifungal activity, high hydrophobicity, mild deacidification, and low-cost facile fabrication.

High-performance archival packaging requires integrated fire, acid, mold, and moisture resistance. Boron-based retardants are ideal modifiers for paper conservation due to their low toxicity, negligible damage to cellulose substrates, high flame-retardant efficiency, low cost, and excellent stability. Borax and disodium octaborate tetrahydrate form compact glassy boron oxide layers at high temperatures to isolate heat and oxygen, realizing condensed-phase flame retardancy [19,20,21,22], while enabling paper deacidification and antifungal protection [23,24]. Nonetheless, pure boron modifiers are intrinsically hydrophilic and prone to leaching in high-humidity environments, causing gradual attenuation of protective performance [25]. In contrast, ODTMS constructs low-surface-energy hydrophobic layers via long alkyl chains and cross-linked siloxane networks, improving paper moisture resistance and inhibiting microbial proliferation [26]. However, a single ODTMS modification cannot impart flame-retardant or antifungal properties to paper.

Herein, a facile two-step complementary coating strategy based on borax–disodium octaborate tetrahydrate (BX-DOT) and octadecyltrimethoxysilane (ODTMS) is proposed for multifunctional paper modification. The incorporated BX-DOT imparts the treated paper with flame retardancy, broad-spectrum antifungal activity, and pH-adjusting deacidification capacity. The subsequent ODTMS spraying constructs a robust hydrophobic interface, effectively addressing the hydrophilic leaching issue inherent to boron-based modifiers. The ODTMS-cross-linked siloxane network firmly anchors and encapsulates boron species on cellulose fiber surfaces. This stepwise fabrication avoids component interference and functional trade-offs commonly observed in one-pot blending systems, enabling the full retention of boron-derived protective performance. Compared with sophisticated nanocomposite preparation routes, this sequential dipping–spraying strategy utilizes low-cost and low-toxicity raw materials. The modified kraft paper integrates excellent flame-retardant, hydrophobic, and antifungal performances, providing a feasible technical route for the long-term safe preservation of paper cultural relics.

## 2. Experiment

### 2.1. Materials

Borax decahydrate (BX, AR) and disodium octoborate tetrahydrate (DOT, Na_2_B_8_O_13_·4H_2_O, 99.5%) were provided by Zhengzhou Ruichang Chemical Products Co., Ltd. (Zhengzhou, China). Hydrochloric acid (HCl) was obtained from Sinopharm Chemical Reagent Beijing Co., Ltd.(Beiging, China). Octadecyltrimethoxysilane (ODTMS, C_21_H_46_O_3_Si) with a purity of 90% (AR) was supplied by Beijing Chemical Works (Beijing, China). All reagents were used as-received without further purification.

### 2.2. Preparation of Flame Retardant Samples

The flame-retardant coating stock solution was prepared by mixing borax (BX) and disodium octaborate tetrahydrate (DOT) at a mass ratio of 2:3 in 100 mL of deionized water. The mixture was magnetically stirred for 1 h to obtain a clear solution. Serial working solutions with concentrations of 0.375, 0.75, 1.5 and 3.0 (*w*/*v*) BX+DOT were further diluted from the stock solution. Kraft paper specimens cut to 2 cm × 4 cm were immersed in the as-prepared flame-retardant solutions to ensure complete wetting. Impregnation was performed at ambient temperature and atmospheric pressure. After 30 min of immersion, the samples were carefully removed with tweezers and air-dried at room temperature to obtain BX+DOT-modified flame-retardant kraft paper. After preparation, all specimens were stored in a dry box (23 ± 2 °C, 45–50% RH), and all characterizations were completed within 48 h. The formulations used for kraft paper treatments are listed in Table 1.

The weight-gain percentage *W-G* was calculated according to the formula: *W-G*$(\%)=(W_{2}-W_{1})/W_{1}\times 100\%$, where *W*_1_ and *W*_2_ represent the dry weights of kraft paper before and after treatment, respectively.

### 2.3. Preparation of Superhydrophobic Flame Retardant Samples

The silica-based sol–gel was synthesized by hydrolysis and condensation of ODTMS in ethanol and water following a reported procedure [27]. A total of 200 µL of ODTMS and 60 mL of anhydrous ethanol were added to a three-necked flask and magnetically stirred for 30 min. Meanwhile, a mixed solution consisting of 18 mL of deionized water, 60 mL of anhydrous ethanol, and 0.5 mL of 0.1 mol/L hydrochloric acid was prepared in advance. This mixed solution was slowly added into the three-necked flask via a dropping funnel over a period of 30 min. After the addition was completed, the reaction was carried out under reflux at 60 °C for 1 h and naturally cooled to room temperature to obtain a homogeneous ODTMS hydrophobic sol solution. The sols aged for 48 h in a closed container at room temperature before spraying.

**Figure 1:**
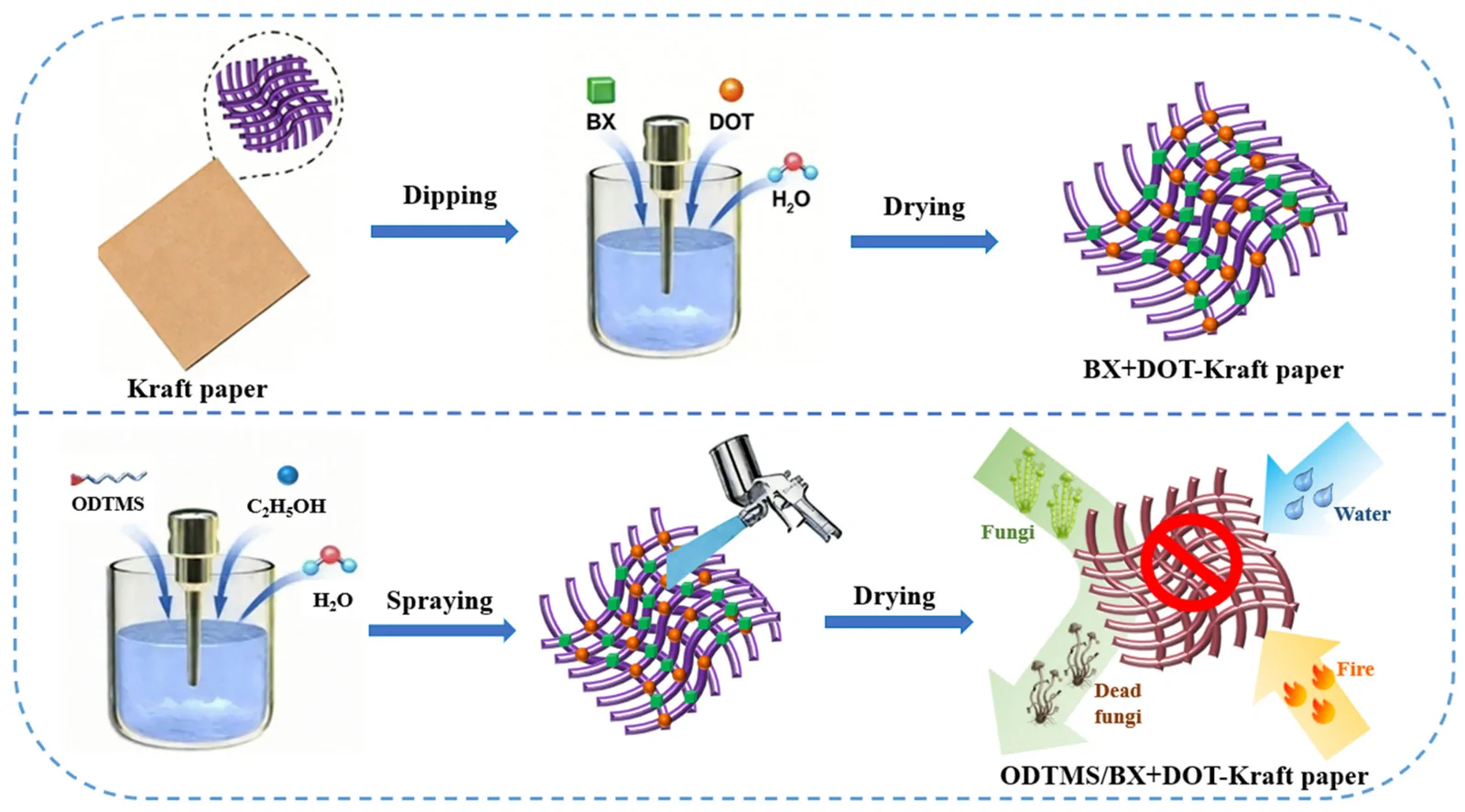
Schematic of hydrophobic flame-retardant kraft paper of preparation process.

The above hydrophobic solution was loaded into a spray gun and applied onto the surface of the pre-treated flame-retardant kraft paper at a pressure of 0.3 MPa. During spraying, the spray gun was maintained at a distance of 20 cm from the substrate and moved back and forth to ensure uniform coverage of the coating. After spraying, the sample was dried and cured in an oven at 100 °C, ultimately yielding superhydrophobic flame-retardant kraft paper. Figure 1 illustrates the preparation process flow of this hydrophobic flame-retardant kraft paper.

### 2.4. Characterizations

#### 2.4.1. Attenuated Total Reflection Fourier Transform Infrared Spectra(ATR-FTIR)

ATR-FTIR spectra of untreated and treated kraft paper were collected using an FTIR instrument (Nicolet iS50, Thermo Fisher Scientific, Waltham, MA, USA). The spectral range and resolution factor of the ATR-FTIR spectrometer were from 4000 to 500^−1^ and 4 cm^−1^, respectively. Every spectrum consisted of 32 scans in the transmission mode.

#### 2.4.2. Thermal Gravimetric Analysis (TGA)

Thermal stability of untreated and treated kraft paper was assessed using a thermogravimetric analyzer (TG 500, Thermon Fisher Scientific, Waltham, MA, USA) under a nitrogen atmosphere. First, we placed 10–15 mg samples into an alumina crucible at a heating rate of 20 °C/min and a temperature range of 25–800 °C, and we recorded the mass loss with changes in temperature. Thermogravimetric analysis (TGA) curves and differential thermogravimetric (DTG) curves were subsequently recorded.

#### 2.4.3. Limiting Oxygen Index (LOI) Test

The LOI test was conducted with an LFY-606B oxygen index meter (Shandong Institute of Textile Science, Jinan, China). The samples were cut to 150mm × 58 mm according to the GB/T5454-1997 [28].

#### 2.4.4. Cone Calorimetry Test (CCT)

Combustion properties of samples were measured using a cone calorimeter (Fire Testing Technology, East Grinstead, UK) with a heat flux of 35 kW/m^2^. The samples (100 × 100 × 0.4 mm^3^) were tested according to the ISO 5660-1 standard [29], and each sample was examined three times.

#### 2.4.5. Scanning Electronic Microscopy (SEM)

The surface micromorpholog of untreated and treated samples and char residues was observed by a SU3500 scanning electron microscope (SEM, Hitachi, Tokyo, Japan) Samples were coated with conductive gold, and the accelerating voltage was set at 10 kV. The attached energy-dispersive spectrometry (EDS) system was applied for elemental analysis.

#### 2.4.6. X-Ray Photoelectron Spectroscopy Measurements (XPS)

The XSAM 800 spectrometer (Kratos Manchester, Manchester, UK) was utilized to test the flame retardant mechanism. XPS was performed with 1486.6 eV Al Kα excitation radiation, operated at 12 kV and 15 mA, and the C 1s line at 284.6 eV was set to the binding energy calibration.

#### 2.4.7. pH Measurement

pH values of the untreated and treated kraft paper samples were measured with a Mettler Toledo Seven Compact^TM^ pH S210 pH meter equipped with a flat electrode (HI1413, Hanna Instruments, Woonsocket, RI, USA).The pH meter was Mettler Toledo, Greifensee, Switzerland.

#### 2.4.8. Contact Angle Test

Water contact angle (WCA) measurements were performed on a Dataphysics OCA 20 contact angle system (DataPhysics Instruments GmbH, Filderstadt, Germany) at ambient temperature. The average WCA value was determined by measuring the WCA at five different positions on the same sample, referring to ISO/TS 14778 [30].

#### 2.4.9. Mold-Proof Test

Eight strains, including *Penicillium citrinum*, *Alternaria alternata*, *Cladosporium* sp., *Trichoderma harzianum*, *Penicillium citrinum*, *Aspergillus flavus*, *Aspergillus niger*, and *Aspergillus fumigatus*, with a concentration of 1 × 10^5^ CFU/mL, were individually cultured on PDA medium. Subsequently, untreated kraft paper and treated kraft paper were placed on the inoculated Petri dishes, respectively, and all Petri dishes were incubated at 28 °C for 48 h. After the incubation period, the antifungal effects of the two types of kraft paper were measured.

## 3. Results and Discussion

### 3.1. ATR-FTIR Spectral Analysis

**Figure 2:**
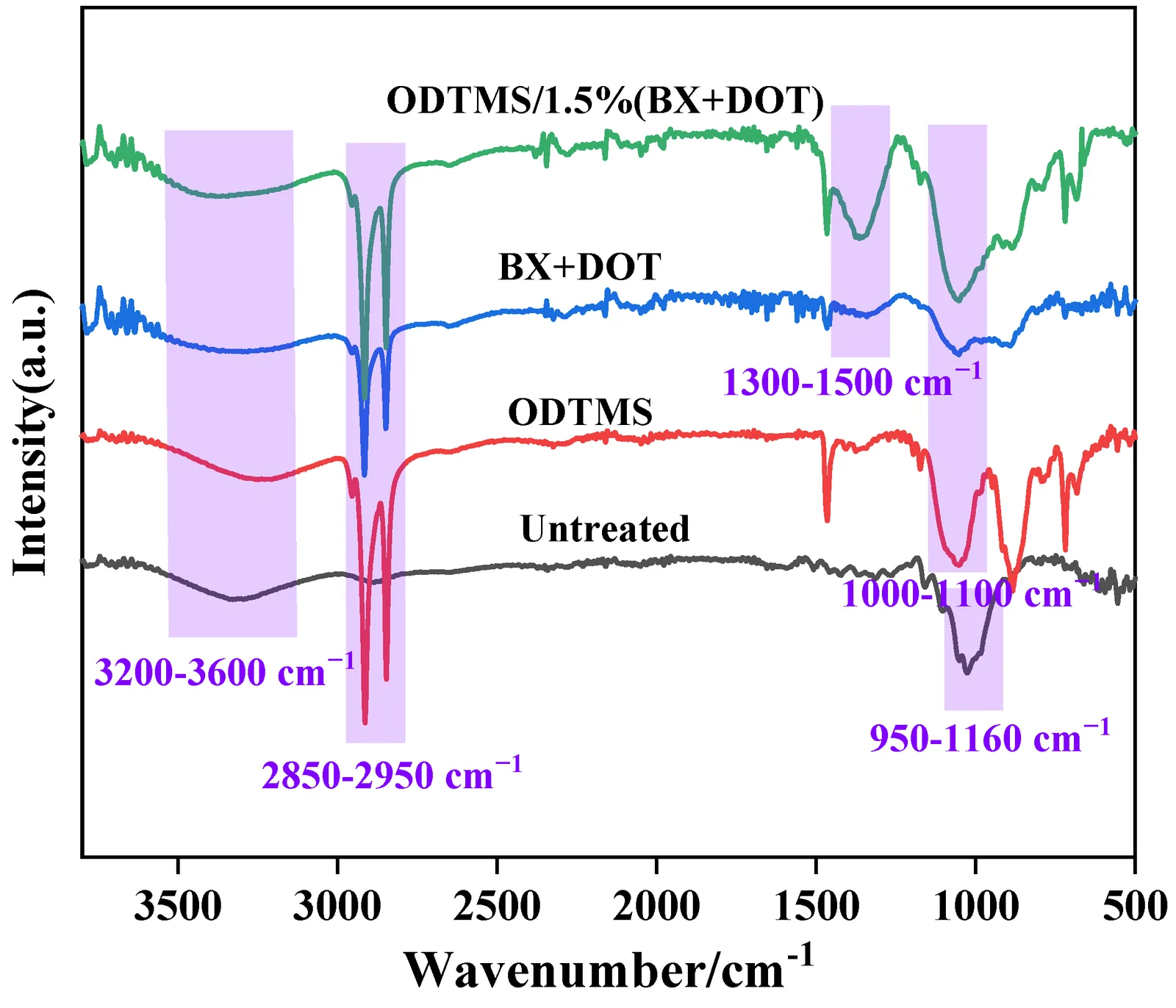
ATR-FTIR spectra of untreated kraft paper and treated kraft paper.

The structures of the untreated and treated kraft papers were determined by Fourier transform infrared spectroscopy (FT-IR) to confirm whether ODTMS, BX+DOT, and their composite modifier were successfully grafted onto the cellulose surface. The FT-IR spectra of the samples are shown in Figure 2. The FT-IR spectrum of the untreated blank sample exhibited the typical characteristics of cellulose. A broad peak at approximately 3340 cm^−1^ was attributed to the stretching vibration of the O–H bonds, a weak peak at ~2900 cm^−1^ was attributed to the stretching vibration of C–H (sp^3^) bonds, and a broad peak in the fingerprint region of 950–1160 cm^−1^ was attributed to the stretching vibrations of C–O–C glycosidic bonds and C–OH skeletons. For the BX+DOT-treated sample, the intensity of the peak at 3340 cm^−1^ decreased due to the interaction between O–H groups and borate. A new characteristic peak corresponding to B–O–B bonds appeared at ~1350 cm^−1^, and the absorption in the 1000–1100 cm^−1^ region was enhanced by the superposition of B–O bond vibrations with those of cellulose functional groups. In the case of the ODTMS-treated sample, characteristic stretching vibration peaks of long-chain alkyl –CH_2_– appeared at 2950 cm^−1^ and 2850 cm^−1^, and a broad, strong absorption band assigned to Si–O–Si was observed in the 1000–1100 cm^−1^ region. Meanwhile, the intensity of the O–H peak at 3340 cm^−1^ was significantly attenuated. These spectral changes reflect the consumption of surface-OH groups upon ODTMS treatment, which may arise from hydrogen-bonding interactions and physical coating deposition. The FT-IR spectrum of the ODTMS/1.5% (BX+DOT)-treated sample exhibited all the characteristic features of both the BX+DOT and ODTMS-treated samples. This spectrum not only retained the characteristic peak of B–O–B bonds at ~1350 cm^−1^ and the superimposed absorption from B–O bond vibrations and cellulose functional groups in the 1000–1100 cm^−1^ region, but also showed the stretching vibration peaks of long-chain alkyl C–H at 2950 cm^−1^ and 2850 cm^−1^, together with the broad strong absorption band of Si–O–Si bonds in the 1000–1100 cm^−1^ region. In addition, further attenuation of the 3340 cm^−1^ O–H band indicated interactions between cellulose fibers and the deposited modifiers.

### 3.2. X-Ray Photoelectron Spectroscopy Analysis

**Figure 3:**
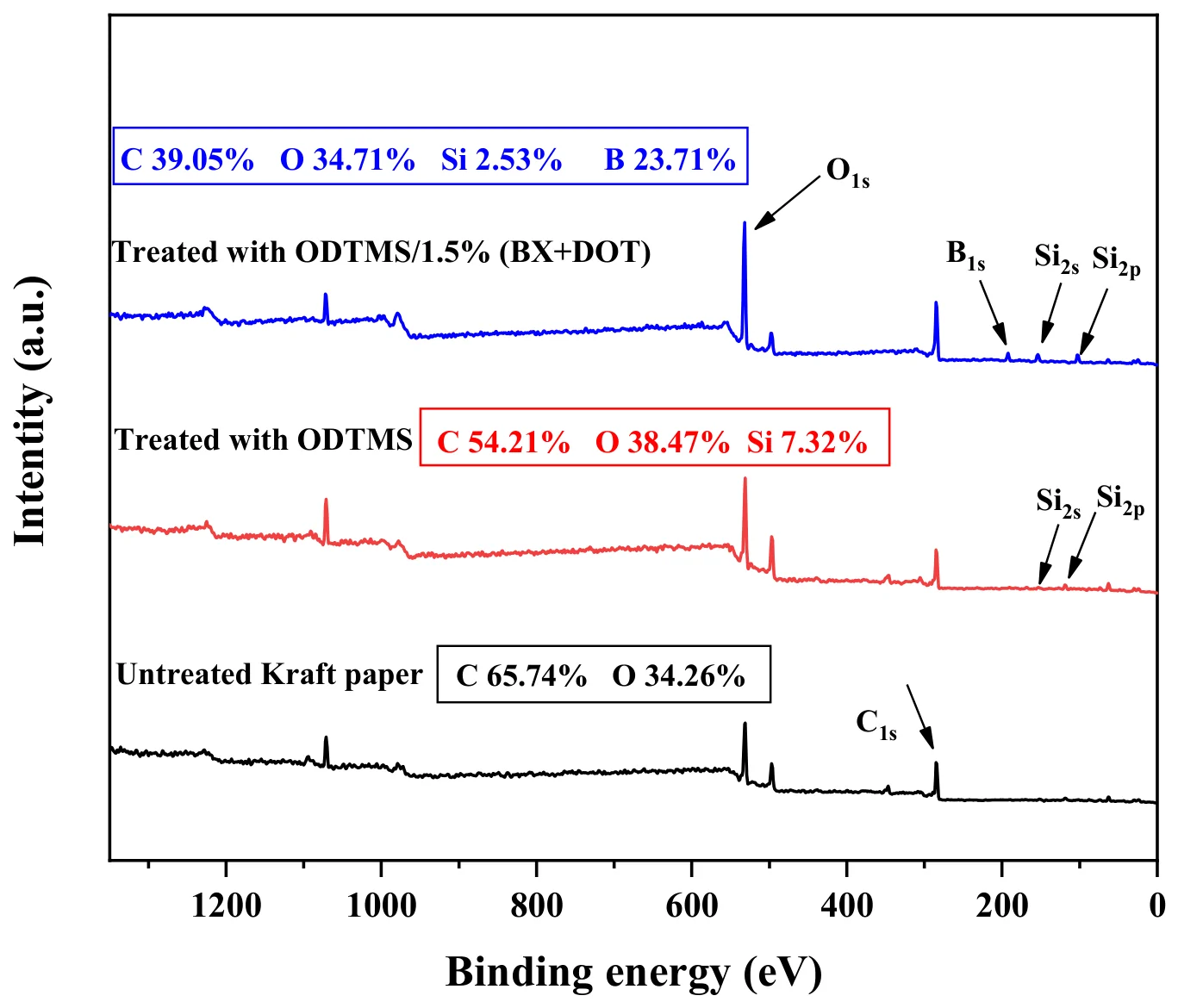
XPS spectra of untreated and treated kraft papers.

X-ray photoelectron spectroscopy was employed to verify the surface chemistry of the kraft papers. Figure 3 shows the XPS spectra of untreated and treated kraft papers. As shown in Figure 3, the surface of the untreated kraft paper was primarily composed of oxygen and carbon, with proportions of 34.26 at.% and 65.74 at.%, respectively. In contrast, for the ODTMS-treated kraft paper, new peaks corresponding to Si_2s_ and Si_2p_ were detected at 103.0 eV and 102.2 eV, respectively [23,24], confirming the successful deposition of ODTMS on the paper surface, with a Si atomic percentage of 7.32%. For the ODTMS/1.5% (BX+DOT)-treated kraft paper, a new peak at 191.44 eV (B1s) was observed, confirming the successful deposition of BX+DOT on the cellulose surface. The surface atomic percentages of Si and B reached 2.53 at.% and 23.71 at.% (Si/B ≈ 0.107). The simultaneous detection of B and Si elements demonstrates that both modifiers are deposited on the composite-treated kraft paper.

### 3.3. pH Values

**Figure 4:**
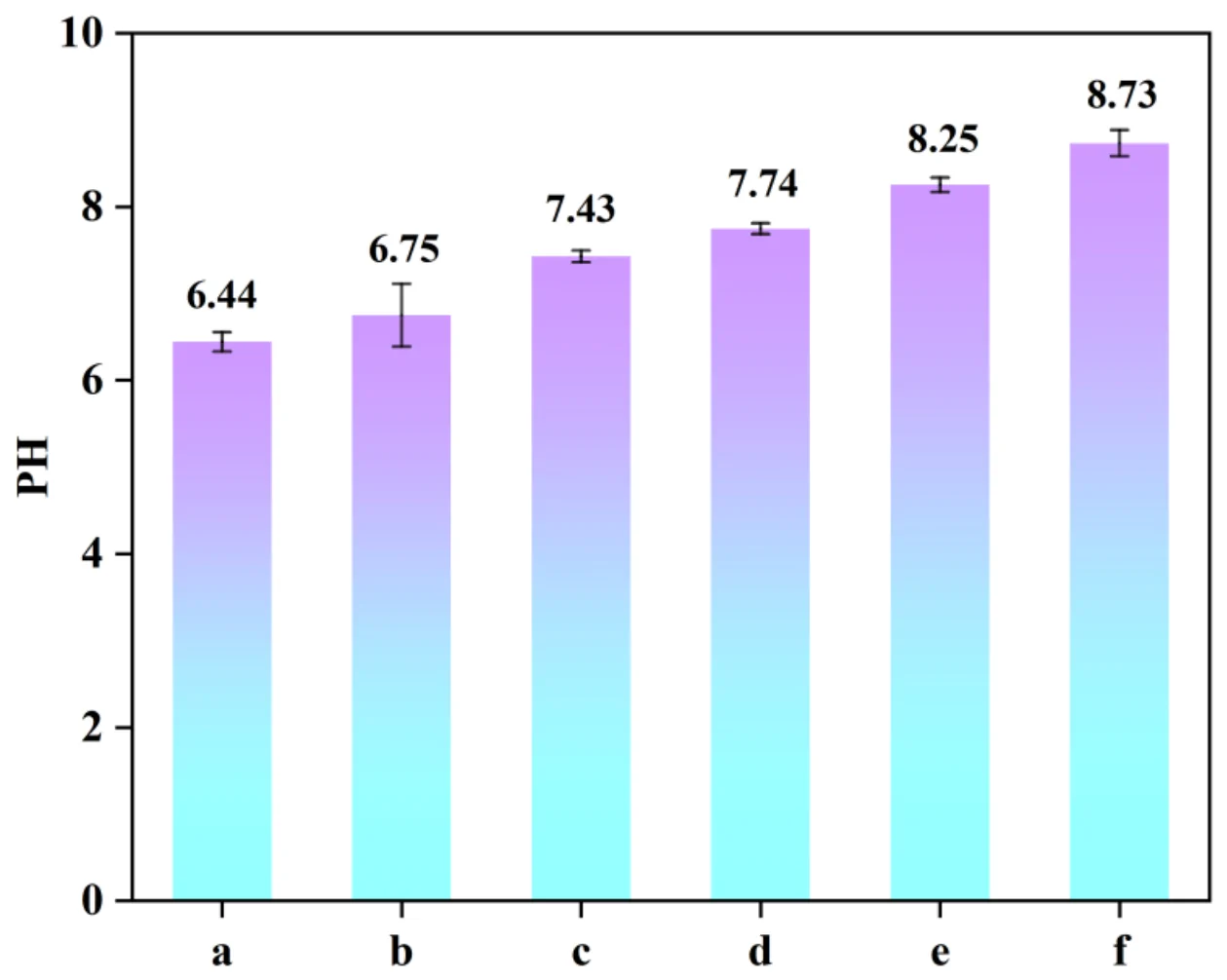
Acidity test results of kraft paper under different treatments: (a) untreated, (b) ODTMS, (c) ODTMS/0.375% (BX+DOT), (d) ODTMS/0.75% (BX+DOT), (e) ODTMS/1.5% (BX+DOT), and (f) ODTMS/3% (BX+DOT).

The pH values of the kraft paper samples under different treatment conditions are shown in Figure 4. The untreated kraft paper (a) exhibited a pH of 6.44, indicating a weakly acidic nature, for unmodified cellulosic materials. Treatment with ODTMS alone (b) resulted in a slight increase in pH to 6.75. With the incorporation of the BX+DOT flame retardant, a pronounced and dose-dependent elevation in pH was observed. As the BX+DOT concentration increased from 0.375% (c) to 3% (f), the pH values rose progressively from 7.43 to 8.73. All composite-coated samples (c–f) displayed pH values above 7, thereby shifting the paper’s property from weakly acidic to weakly alkaline. This significant increase is attributed to the alkaline nature of the BX+DOT flame retardant, which introduces basic functional groups that effectively neutralize the inherent acidity of cellulose. Meanwhile, the presence of residual alkaline particles enhances the resistance of cellulosic materials to acid-catalyzed degradation, thereby improving their long-term stability and durability.

### 3.4. Water Contact Angle (WCA) Measurements

**Figure 5:**
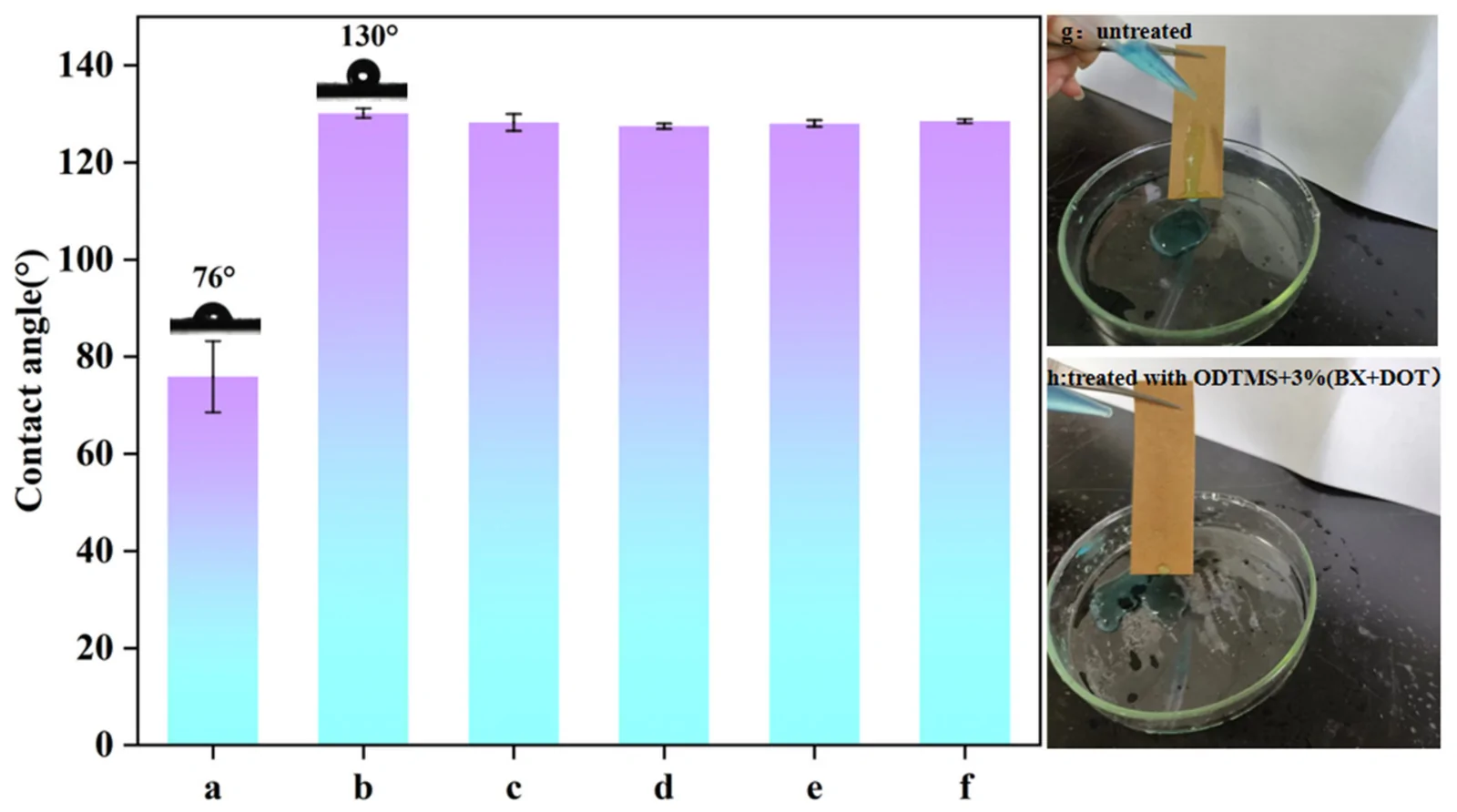
Water contact angle measurements of kraft paper under different treatments: (a) untreated, (b) ODTMS, (c) ODTMS/0.375% (BX+DOT), (d) ODTMS/0.75% (BX+DOT), (e) ODTMS/1.5% (BX+DOT), and (f) ODTMS/3% (BX+DOT).

Surface wettability is an important property of a solid surface. To determine the degree of surface modification, the water contact angle (WCA) of a droplet on the solid surface was measured using water as the test liquid [31]. The WCAs of the kraft paper samples under different treatment conditions are shown in Figure 5. The untreated kraft paper (a) exhibited a WCA of 76°, indicating a hydrophilic nature. In sharp contrast, all samples treated with ODTMS (b–f) showed a marked increase in WCAs, with values exceeding 130°, clearly demonstrating the successful fabrication of hydrophobic surfaces. Notably, the incorporation of the (BX+DOT) flame retardant did not compromise the hydrophobicity of the coatings. All composite-coated samples (c–f) maintained WCAs above 130°, suggesting that (BX+DOT) was encapsulated on the fiber surface by ODTMS. The untreated sample (g) readily absorbed water, whereas the treated sample (h) effectively repelled water droplets, forming distinct, nearly spherical beads on its surface, further validating the hydrophobic nature of the modified paper. The observed hydrophobicity originates from the low-surface-energy long alkyl chains of ODTMS [32], together with the hierarchical micro/nanoscale roughness generated during the coating process. The low-surface-energy component and surface roughness jointly inhibit water penetration, endowing the paper with improved water-repellent capacity.

### 3.5. Charring Behavior of Untreated and Treated Kraft Paper

A cone calorimeter is a widely used apparatus for evaluating the combustion behaviors of various polymeric materials under simulated fire conditions. To examine the combustion behaviors of the untreated and treated papers, cone calorimeter tests were conducted [33].

**Figure 6:**
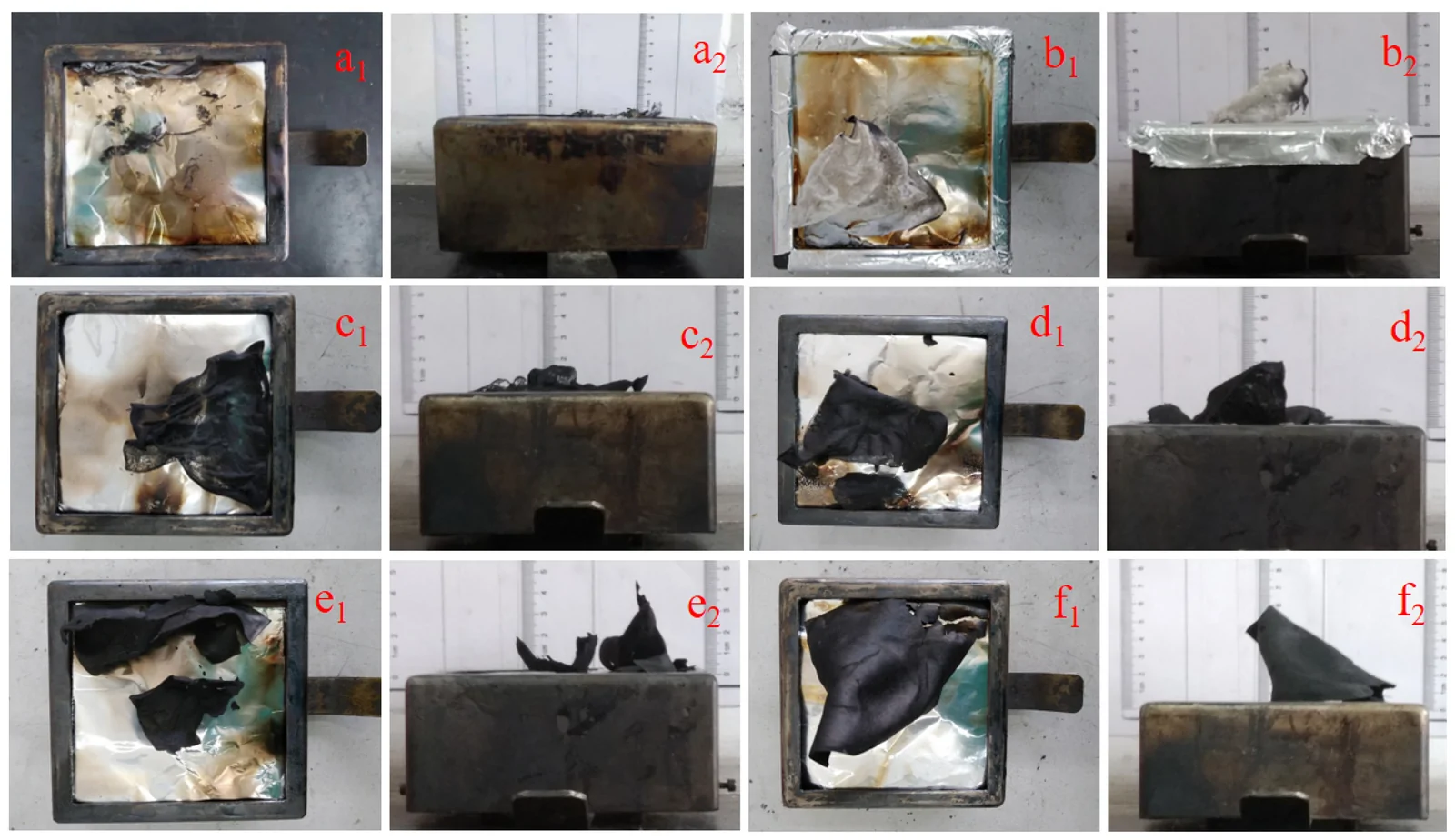
Digital photographs of chars residues of kraft paper with different treatments after the cone calorimeter test: (**a**) untreated, (**b**) ODTMS, (**c**) ODTMS/0.375% (BX+DOT), (**d**) ODTMS/0.75% (BX+DOT), (**e**) ODTMS/1.5% (BX+DOT), and (**f**) ODTMS/3% (BX+DOT) treated samples, respectively. Subscripts _1_ and _2_ denote top view and side view of char residues.

Figure 6 presents the digital images of the char residues. From Figure 6a, it can be observed that the untreated kraft paper burned out, leaving minimal white ashes. This indicates that the untreated paper undergoes almost complete decomposition during combustion. In contrast, the ODTMS-treated sample (Figure 6b) formed a thin, continuous char film, and the char integrity was further improved with the addition of BX+DOT. As the BX+DOT loading increased from 0.375% to 3% (Figure 6c–f), the residual char became progressively thicker, denser, and more intact. Notably, the sample treated with ODTMS/3% (BX+DOT) (Figure 6f) exhibited the most robust and compact char layer, which acted as a highly effective physical barrier to inhibit heat and mass transfer during combustion. This synergistic char formation between ODTMS and BX+DOT significantly enhances the flame retardancy of the kraft paper.

**Figure 7:**
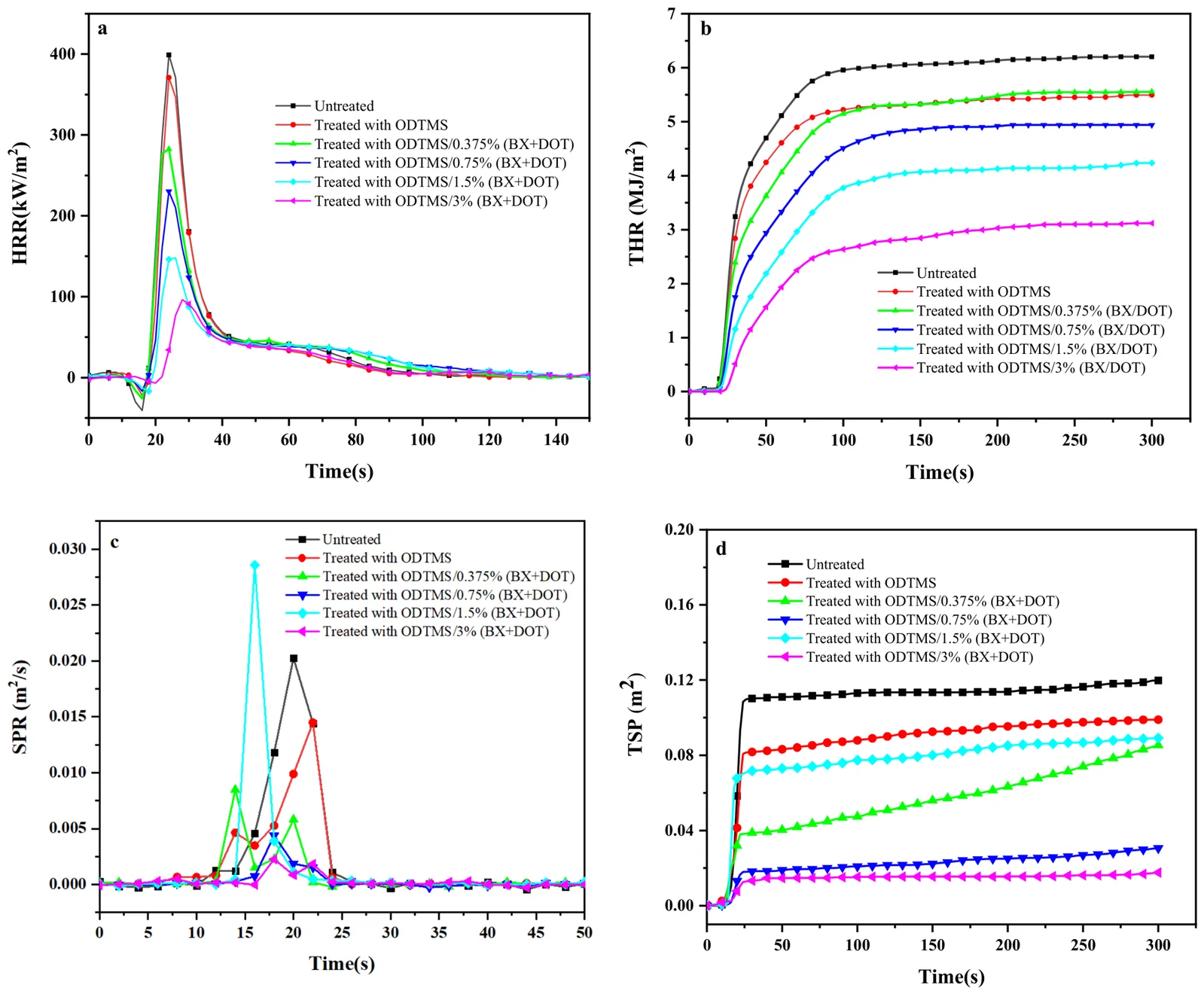

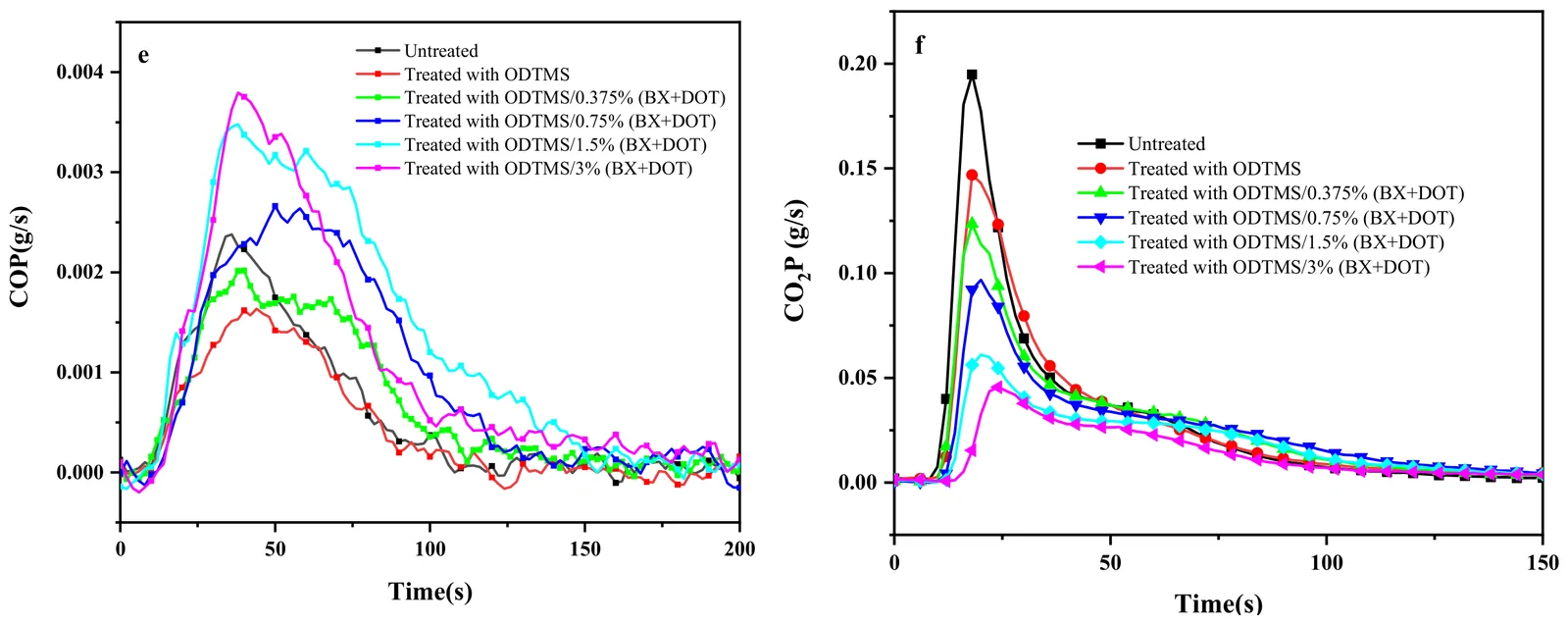
Plots of HRR (**a**), THR (**b**), SPR (**c**), TSP (**d**), COP (**e**), and CO_2_P (**f**) curves of the control untreated and treated kraft papers composites with combustion time obtained at 35 kW/m^2^ from cone calorimetry.

The heat release rate (HRR), total heat release (THR), mass loss, total smoke production (TSP), smoke production rate (SPR), and CO production (COP) curves of the untreated and treated kraft papers are shown in Figure 7, and some key data were recorded in Table 2. In Figure 7a, the peak heat release rate (PHRR) values of the treated samples were 370.6, 283.0, 228.7, 145.1, and 96.15 kW/m^2^, respectively, which are significantly lower than the untreated kraft paper (398.2 kW/m^2^). Moreover, a higher content of BX and DOT resulted in a better flame-retardant effect. The total heat release (THR) curves of the treated samples showed similar trends; the THR values for the treated samples (5.5, 5.4, 4.9, 4.2, and 3.0 MJ/m^2^, respectively) were lower than that for the untreated kraft paper (6.2 MJ/m^2^) (Figure 7b). The PHRR and THR are important determinants for evaluating the combustion performance of paper. The reduced PHRR and THR values demonstrate that the modifier can suppress the heat release intensity and total heat output during combustion. ODTMS/BX+DOT effectively improved the flame retardancy of kraft paper.

The smoke production rate (SPR) and total smoke production (TSP) profiles of the kraft paper samples are displayed in Figure 7c,d. The untreated sample exhibited an SPR peak of 0.020 m^2^/s at 15 s and a final TSP of 0.12 m^2^, indicating intense smoke release. In contrast, all modified samples showed significantly reduced SPR peaks and TSP values in a dose-dependent manner. Notably, the sample treated with ODTMS/3 g·mL^−1^ (BX+DOT) exhibited the most stable SPR curve and the lowest final TSP of 0.03 m^2^. This can be attributed to the formation of a dense, protective SiO_2_-B_2_O_3_ char layer during combustion, which effectively hinders heat and mass transfer, thereby suppressing smoke generation and release.

The CO release rate (COP) and CO_2_ release rate (CO_2_P) profiles of the kraft paper samples under different treatment conditions are shown in Figure 7e,f. For the COP curves (Figure 7e), the untreated kraft paper exhibited a distinct peak of approximately 0.0023 g/s during the initial combustion stage (20–40 s), indicating significant generation of toxic CO from incomplete combustion. In contrast, all modified samples showed a notable reduction in COP, with the peak value decreasing and the peak time being delayed as the BX+DOT loading increased, indicating a progressive suppression of incomplete combustion by the flame-retardant system.

Regarding the CO_2_P curves (Figure 7f), the untreated kraft paper displayed an extremely high peak of 0.19 g/s within the first 10 s of combustion, reflecting intense and rapid burning. All modified samples exhibited significantly reduced CO_2_P peaks in a dose-dependent manner: the peak values decreased to 0.12 g/s, 0.10 g/s, 0.06 g/s, and 0.04 g/s for samples with 0.375%, 0.75%, 1.5%, and 3% (BX+DOT) loadings of 0.375%, 0.75%, 1.5%, and 3%, respectively. Moreover, the peak appearance time was progressively delayed with increasing flame-retardant content, indicating that the combustion reaction was effectively retarded. The synergistic flame-retardant effect of ODTMS and BX+DOT can be attributed to the formation of a dense char layer composed of SiO_2_ (from ODTMS) and B_2_O_3_ (from BX+DOT decomposition) on the fiber surface during combustion [34,35]. This layer acts as a physical barrier, inhibiting heat and oxygen transfer, thereby suppressing the combustion process and reducing the release of both toxic and flue gases.

**Figure 8:**
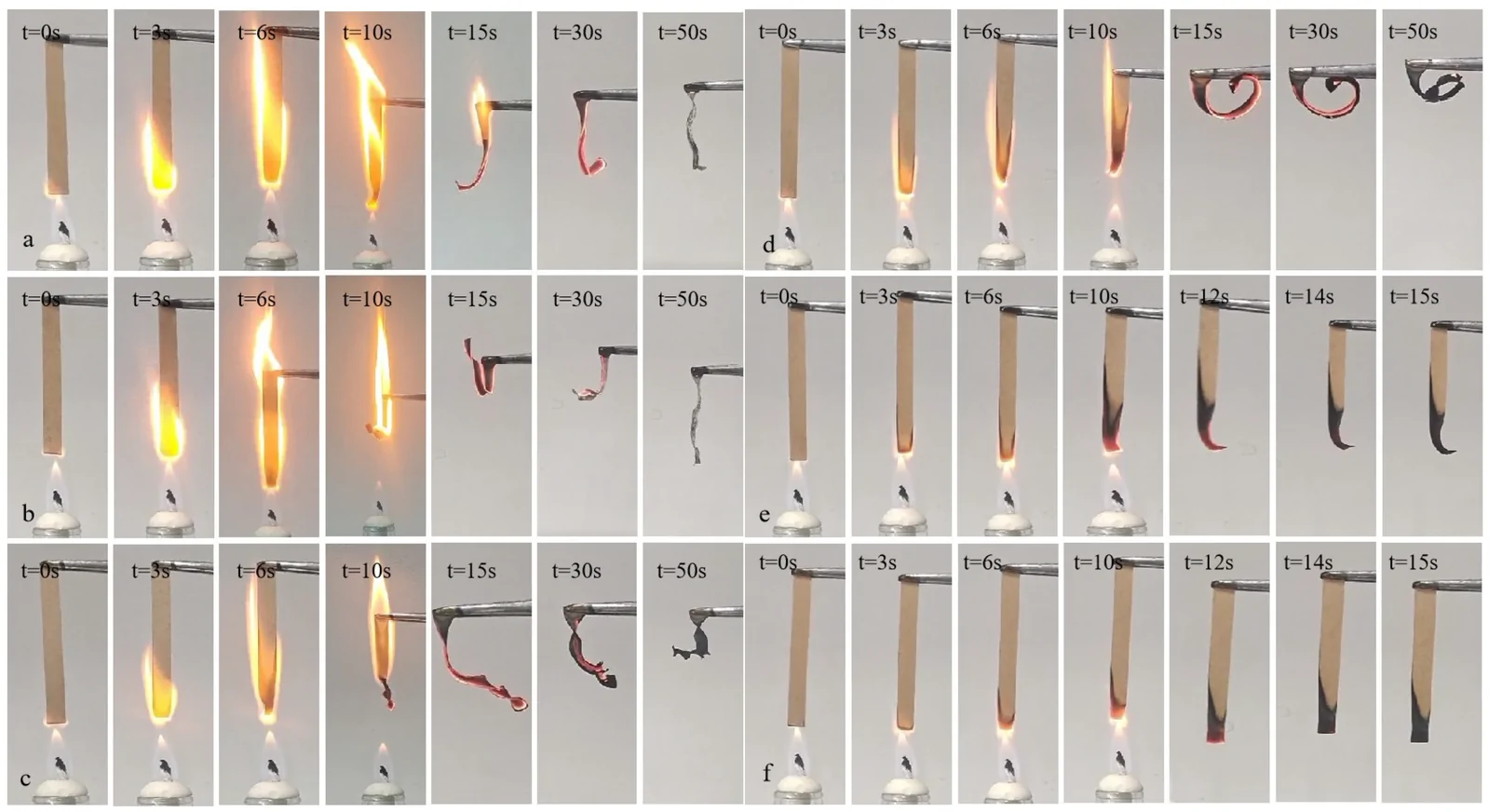
Digital photographs of differently treated kraft paper samples in the vertical burning test: (**a**) untreated, (**b**) ODTMS, (**c**) ODTMS/0.375% (BX+DOT), (**d**) ODTMS/0.75% (BX+DOT), (**e**) ODTMS/1.5% (BX+DOT), and (**f**) ODTMS/3% (BX+DOT).

In this work, vertical burning tests and the LOI instrument were employed to assess the flame-retardant performance of the untreated and treated kraft paper, providing pivotal evidence for evaluating their application value. Figure 8 presents the dynamic combustion behavior of the kraft paper samples with different treatments during vertical burning tests, offering direct visual evidence of their flame-retardant performance. The untreated kraft paper (Figure 8a) ignited and burned vigorously immediately after exposure to the flame, with a large, intense flame reaching its maximum height within 10 s. The flame persisted for a prolonged duration, remaining visible for over 15 s and requiring more than 30 s to self-extinguish completely. After combustion, only a small quantity of loose, curled, and fragile ash remained, with no intact char structure. This behavior confirms that the untreated paper is highly flammable, exhibits rapid flame propagation, lacks self-extinguishing capability, and possesses extremely poor flame retardancy. In contrast, the ODTMS-treated sample (Figure 8b) showed a noticeable reduction in flame height and combustion intensity, with self-extinguishment occurring at approximately 15 s. The amount of residual char increased, and the degree of curling was reduced, although the char layer remained thin and fragile, and exhibited visible shrinkage and breakage, indicating that ODTMS alone provides only limited protection. With the incorporation of BX+DOT, a clear dose-dependent improvement in flame retardancy was observed. As the (BX+DOT) loading increased from 0.375% to 3%, the flame height, combustion intensity, and self-extinguishing time all decreased progressively. Notably, the sample with ODTMS/3% (BX+DOT) exhibited the most stable combustion behavior: only slight charring was observed at the bottom of the sample, no obvious flame was sustained, and complete self-extinguishment was achieved within 15 s. The resulting char layer was the thickest, densest, and most intact among all samples, with minimal curling or shrinkage, effectively acting as a physical barrier to prevent further flame spread.

The LOI and vertical burning test results are summarized in Table 3. The untreated kraft paper exhibited an LOI of 20.6 ± 0.2%, a flame time of 16 ± 2 s, an afterglow time of 182 ± 3 s, and a damaged length of 210 mm, indicating high flammability. In contrast, the treated sample showed a markedly enhanced LOI of 37.5 ± 0.3%, a drastically reduced afterflame time of 1 ± 0.3 s, a shortened afterglow time of 27 ± 1 s, and a negligible damaged length of 3.0 ± 2 mm. These results confirm that the ODTMS/boron-based flame-retardant treatment effectively transforms kraft paper from a highly flammable material into a flame-retardant material with excellent self-extinguishing ability and minimal flame spread.

### 3.6. Analyses of Thermal Stability of Untreated and Treated Kraft Paper

**Figure 9:**
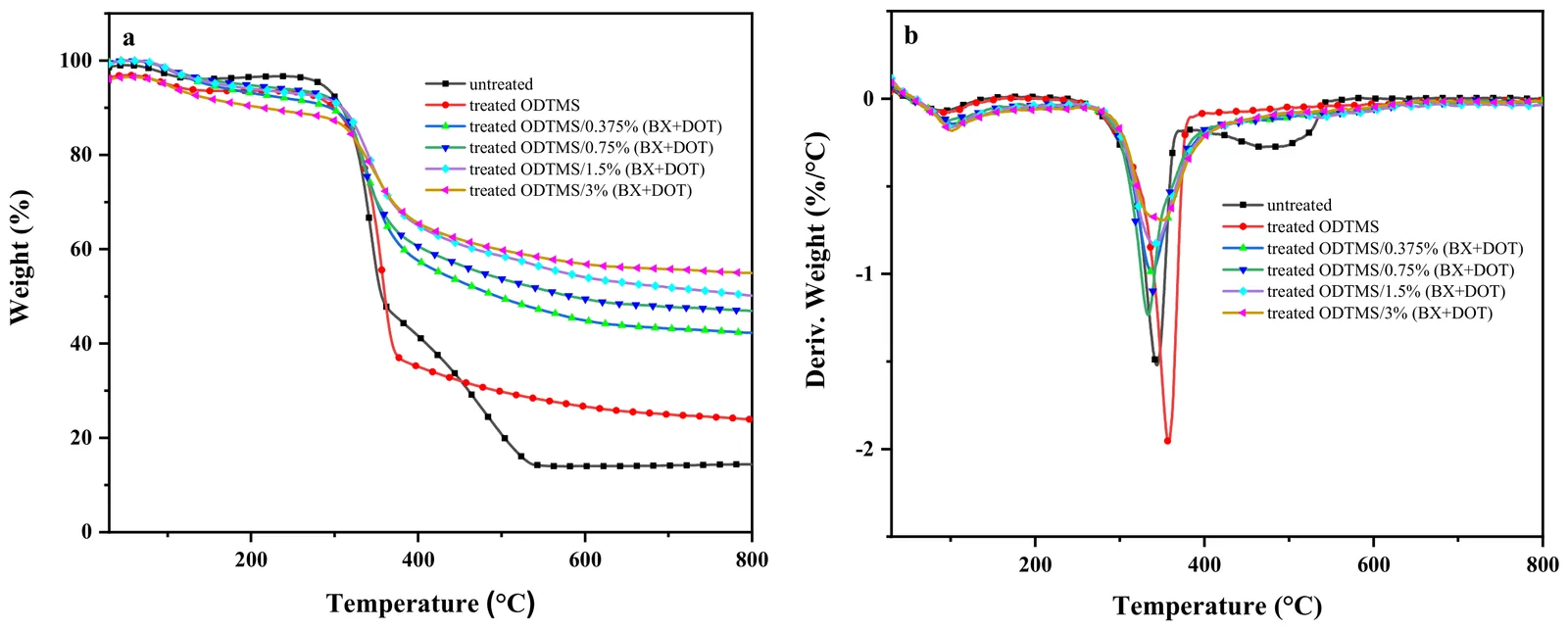
TG (**a**) and DTG (**b**) curves of the untreated and treated papers under N_2_.

The pyrolysis behaviors of the untreated and treated kraft paper samples under a nitrogen atmosphere were investigated by thermogravimetric analysis (TG) and derivative thermogravimetry (DTG) (Figure 9), and the corresponding main pyrolysis parameters are summarized in Table 4.

From Figure 9a, it can be observed that the pyrolysis processes of all samples can be divided into three distinct stages: (1) an initial stage (30–200 °C), (2) a rapid pyrolysis stage (200–400 °C), and (3) a final stage (400–750 °C). In the initial stage, physically adsorbed water and small-molecule additives are released, with mass loss not exceeding 10.0%. In the rapid pyrolysis stage, moisture in the coatings is completely volatilized, followed by coating cracking, fragmentation, and decomposition. This exposes the cellulose fibers to heat, leading to their decomposition and the release of volatiles. The formed char residues construct a continuous flame-retardant shell, which slows the pyrolysis rate during this rapid pyrolysis stage. In the final stage, the residual char undergoes further pyrolysis, with a mass loss below 10.0% and stable final residues. Untreated kraft paper shows poor thermal stability (T_i_ = 200 °C, T_max_ = 320 °C, and a char residue of 14.28% at 750 °C). ODTMS treatment moderately improves stability (T_i_ = 210 °C, T_max_ = 335 °C, and a char residue of 24.44%) by forming a dense organic barrier. With increasing BX+DOT content, the thermal stability is further enhanced; the ODTMS/3% (BX+DOT) sample achieves the highest T_i_ (225 °C), T_max_ (350 °C), and char residue (55.37%), reflecting the favorable modification effect of composite treatment.

Mechanistically, silicon-based additives are known to form thermally stable char layers that inhibit heat and volatile transfer and improve filler dispersion during combustion [36,37]. Accordingly, ODTMS in this work is presumed to generate silicon-containing protective char layers, which suppress thermal decomposition of cellulose. Boron-based flame retardants typically undergo endothermic dehydration and produce inorganic boron-rich species to promote char formation and oxygen isolation [38,39,40,41]. Consistent with the improved thermal stability observed in TG tests, the BX+DOT system may generate boron-containing components that accelerate cellulose carbonization and stabilize residual char. The improved thermal stability and flame retardancy of modified kraft paper are attributed to the individual reinforcing effects of ODTMS and BX+DOT components.

### 3.7. Analyses of Surface Microscopic Morphologies of Char Residues

**Figure 10:**
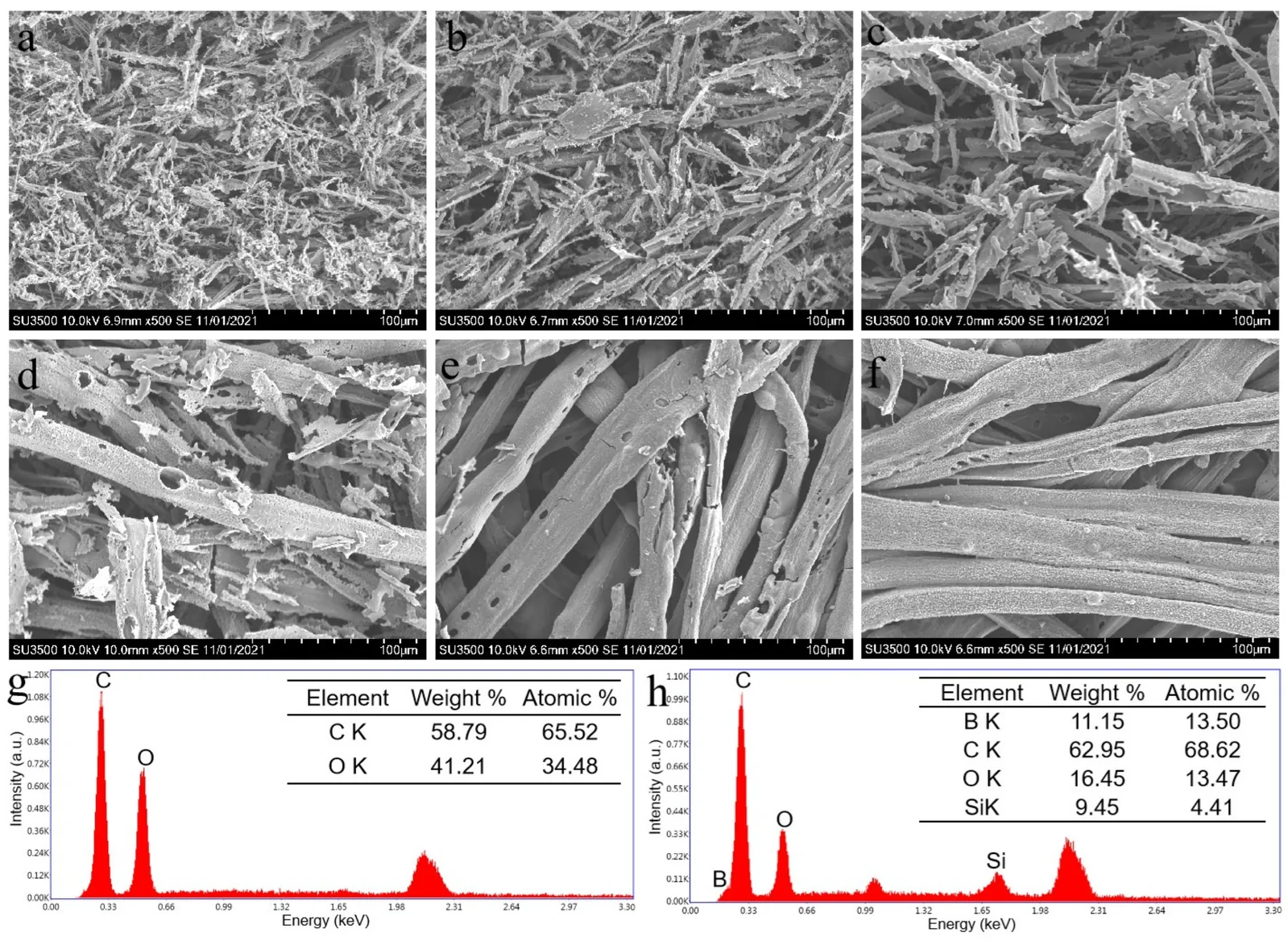
SEM micrographs of char residues after combustion of the pristine and treated kraft papers; (**a**) pristine kraft paper, (**b**) ODTMS-treated kraft paper, (**c**) ODTMS/0.375% (BX+DOT), (**d**) ODTMS/0.75% (BX+DOT), (**e**) ODTMS/1.5% (BX+DOT), and (**f**) ODTMS/3% (BX+DOT). EDS spectra: (**g**) pristine kraft paper, (**h**) ODTMS/3% (BX+DOT)-treated kraft paper.

The surface microscopic morphologies of the char residues for the untreated and treated kraft papers were characterized by SEM, as shown in Figure 10. For the pristine kraft paper (Figure 10a), the fiber structure is loose and porous, showing obvious collapse and fracture of the fiber skeleton, with almost no continuous protective layer. The produced char layer is naturally formed by the dehydration of the D-anhydroglucose ring units in the fibers [42]. This porous structure cannot effectively block heat and oxygen, leading to the rapid release of combustible volatiles during combustion and the rapid spread of the flame. For the ODTMS-treated kraft papers, the residue (Figure 10b) retained the fiber structure with partial damage. With increasing BX+DOT content (Figure 10c,f), the char layer becomes progressively denser and more continuous. Low loadings (0.375–0.75%) result in reduced porosity and less fiber breakage, while high loadings (1.5–3%) form a compact, glassy char layer that fully encapsulates the fibers, eliminating most pores and fractures. This dense, continuous char layer acts as a robust physical barrier to heat and oxygen. EDS analysis confirms the presence of B and Si in the modified char residues. According to previously reported thermal decomposition characteristics of boron- and silicon-based flame retardants, it is speculated that BX+DOT and ODTMS may decompose to produce boron- and silicon-containing inorganic species during combustion. Such inorganic species may melt and condense on fiber surfaces to strengthen the protective char layer and hinder the transfer of heat and oxygen, improving the flame-retardant performance of modified kraft paper.

### 3.8. Antifungal Tests of Kraft Paper

**Figure 11:**
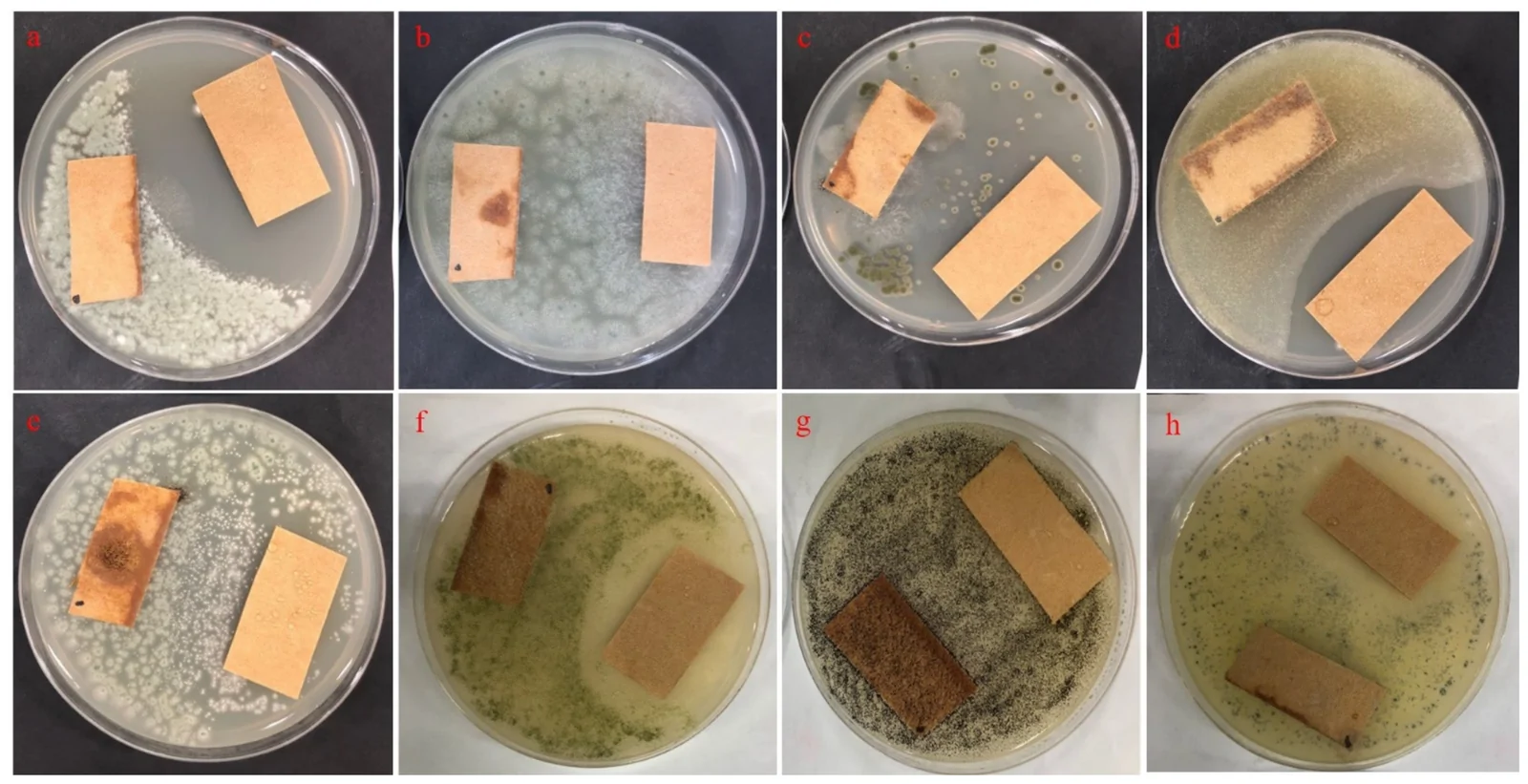
Antifungal test results of untreated kraft paper and treated kraft papers: (**a**) *Penicillium citrinum*, (**b**) *Alternaria alternata*, (**c**) *Cladosporium* sp., (**d**) *Trichoderma harzianum*, (**e**) *Penicillium citrinum*, (**f**) *Aspergillus flavus*, (**g**) *Aspergillus niger*, (**h**) *Aspergillus fumigatus*.

Cellulose-based materials such as kraft paper are highly susceptible to fungal growth and mildew under warm and humid conditions, which seriously reduces their mechanical strength, appearance, and service life. Therefore, it is of great practical significance to evaluate the antifungal properties of the modified paper. The results of the antifungal test are shown in Figure 11, where each Petri dish contains an untreated kraft paper sample on the left and a modified kraft paper sample (ODTMS/3% (BX+DOT)) on the right. The modified kraft paper exhibited varying antifungal activity against different fungi. It showed the strongest inhibitory effect against *Penicillium citrinum*, *Trichoderma harzianum*, and *Aspergillus flavus*; moderate activity against *Aspergillus niger*, *Aspergillus fumigatus*, and *Cladosporium* sp.; and relatively weak activity against *Alternaria alternata*. The antifungal mechanism is mainly attributed to the borate ions dissociated from the BX+DOT system. These ions act as weak Lewis acids and specifically bind to biomolecules with adjacent hydroxyl groups in microbial cells, such as enzymes, nucleic acids, and cell membrane lipids. By inhibiting enzyme activity, disrupting cell membrane integrity, interfering with nucleic acid synthesis, and suppressing spore germination, they inhibit the growth and reproduction of microorganisms or cause their death. The broad-spectrum antifungal properties of the modified kraft paper effectively solve the problem of easy mildew growth in traditional cellulose materials under humid environments, markedly enhancing its application potential and service life as a packaging material in the field of cultural relic protection.

## 4. Conclusions

This paper addresses the problems of fungal erosion, flammability, and moisture-induced degradation of paper archives. Kraft paper for archival packaging was modified with BX+DOT (boron-based flame retardants) and ODTMS (silane-based hydrophobic agent) via a two-step strategy to fabricate multifunctional paper with flame-retardant, hydrophobic, and antifungal properties. The structure, performance, and modification mechanism were systematically investigated. The results demonstrate that the ODTMS/BX+DOT system markedly improves the comprehensive performance of kraft paper. BX+DOT serves as the core functional component providing excellent flame retardancy, antifungal activity, and mild deacidification capacity for paper substrates. ODTMS imparts hydrophobicity to offset the inherent hydrophilic drawback of boron-based modifiers. The limiting oxygen index (LOI) of the modified paper increases from 20.6% to 37.5%, with an afterflame time of ≤1 s and a damaged length of 3.0 mm. The char residue at 750 °C increases from 14.28% for pristine kraft paper to 55.37% for the treated samples, exerting condensed-phase barrier flame-retardant effects, which are supported by LOI, vertical burning and TGA test data. Meanwhile, ODTMS forms a stable organosilicon network structure on the fiber surface, endowing the modified paper with prominent hydrophobicity (water contact angle ≈ 130°). The treated kraft paper shows obvious inhibitory effects against eight fungi strains, especially *Penicillium citrinum* and *Aspergillus flavus*, which are mainly attributed to borate ions disrupting microbial metabolic processes. This facile two-step dipping–spraying modification strategy realizes synchronous multi-protection of kraft paper and overcomes the functional limitations of conventional archival packaging materials. This work provides promising material and a technical route for the long-term preservation of paper archives and cultural relic conservation.

## Figures and Tables

**Table 1:** Formulations of kraft paper samples.

Sample	BX+DOT		*W-G*(%)
	BX (mg·mL^−1^)	DOT (mg·mL^−1^)	
Control kraft paper	0	0	-
0.375% (BX+DOT)	1.50	2.25	8.0 ± 0.8
0.75% (BX+DOT)	3.00	4.50	18.2 ± 1.4
1.5% (BX+DOT)	6.00	9.00	27.9 ± 1.8
3.0% (BX+DOT)	12.00	18.00	42.0 ± 0.5

**Table 2:** Data obtained from CCT for the untreated and treated papers.

Sample	PHRR (kW/m^2^)	THR (MJ/m^2^)	Peak-SPR (m^2^/s)	TSP (m^2^)	Peak-COP (g/s)	Peak-CO_2_P (g/s)
Untreated	398.2	6.2	0.02	0.12	0.0023	0.19
ODTMS	370.6	5.5	0.0145	0.091	0.0018	0.12
ODTMS/0.375% (BX+DOT)	283	5.4	0.0087	0.072	0.0014	0.10
ODTMS/0.75% (BX+DOT)	228.7	4.9	0.0058	0.058	0.0011	0.06
ODTMS/1.5% (BX+DOT)	145.1	4.2	0.0288	0.044	0.0008	0.04
ODTMS/3% (BX+DOT)	96.15	3	0.0021	0.03	0.0005	0.03

**Table 3:** The LOI and vertical burning test results.

Samples	LOI (%)	Afterflame (s)	Afterglow Time (s)	Damaged Length (mm)
Untreated	20.6 ± 0.2	16 ± 2	182 ± 3	210
Treated	37.5 ± 0.3	1 ± 0.3	27 ± 1	3.0 ± 2

**Table 4:** TG data of kraft paper samples under nitrogen atmosphere.

Samples	T_i_ (°C)	T_f_ (°C)	Temperature Difference (T_f_ − T_i_, °C)	T_max_ (°C)	Char Residue Rate (%) 750 °C
Untreated	200	350	150	320	14.28
ODTMS	210	370	160	335	24.44
Treated ODTMS/0.375% (BX+DOT)	215	380	165	340	42.77
Treated ODTMS/0.75% (BX+DOT)	220	390	170	345	47.40
Treated ODTMS/1.5% (BX+DOT)	223	399	172	348	51.12
Treated ODTMS/3% (BX+DOT)	225	400	175	350	55.37

Note: T_i_ is the initial pyrolysis temperature when the sample mass loss reaches 10.0%, °C; T_f_ is the extrapolated offset temperature which corresponds to the end of the sample pyrolysis process, °C; T_max_ is the temperature at which the maximum mass loss occurs, °C.

## Data Availability

The original contributions presented in this study are included in the article. Further inquiries can be directed to the corresponding author.
